# Psychometric Analysis and Cross-Cultural Adaptation of the Croatian Version of the Oral Health Values Scale (OHVS)

**DOI:** 10.3390/dj13020056

**Published:** 2025-01-27

**Authors:** Katarina Major Poljak, Ivana Barać, Ingrid Kovačević, Karla Rožac, Meri Reili, Robert Lovrić, Željko Mudri, Slavko Čandrlić, Marija Čandrlić

**Affiliations:** 1 Department of Integrative Dental Medicine, Faculty of Dental Medicine and Health Osijek, J.J. Strossmayer University of Osijek, Crkvena 21, 31 000 Osijek, Croatia; katarina.major6@gmail.com (K.M.P.); kovacevic.ingrid@gmail.com (I.K.); 2 Department of Nursing and Palliative Medicine, Faculty of Dental Medicine and Health Osijek, J.J. Strossmayer University of Osijek, Car Hadrijan Street 10e, 31 000 Osijek, Croatia; ivana.barac@fdmz.hr (I.B.); zmudri@fdmz.hr (Ž.M.); 3 Department of Anatomy, Histology, Embryology, Pathological Anatomy and Pathological Histology, Faculty of Dental Medicine and Health Osijek, J.J. Strossmayer University of Osijek, Crkvena 21, 31 000 Osijek, Croatia; karla.rozac@fdmz.hr; 4 Interdisciplinary University Study of Molecular Biosciences, J.J. Strossmayer University of Osijek, Trg Sv. Trojstva 3, 31 000 Osijek, Croatia; 5 Department of Integrative Medicine, Faculty of Dental Medicine and Health Osijek, J.J. Strossmayer University of Osijek, Bana Josipa Jelačića 19A, 33 515 Orahovica, Croatia; mreili@fdmz.hr; 6 Doctoral Study in Educational Sciences and Perspectives on Education, Faculty of Education, J.J. Strossmayer University of Osijek, Car Hadrijan Street 10, 31 000 Osijek, Croatia; 7 Nursing Institute “Professor Radivoje Radić”, Faculty of Dental Medicine and Health Osijek, J.J. Strossmayer University of Osijek, Car Hadrijan Street 10 E, 31 000 Osijek, Croatia; rlovric@fdmz.hr; 8 Department of Sociology, Croatian Chatolic University, Ilica Street 242, 10 000 Zagreb, Croatia; 9 Department of Interdisciplinary Area, Faculty of Dental Medicine and Health Osijek, J.J. Strossmayer University of Osijek, Crkvena 21, 31 000 Osijek, Croatia

**Keywords:** health values, oral health, scale validation

## Abstract

**Background/Objectives:** A person’s values regarding oral health significantly shape their related behaviors and habits. Therefore, it is essential to systematically study this relationship and create reliable tools to assess perceptions of oral health values, which can inform evidence-based interventions and policy decisions. This study aimed to translate, culturally adapt, and validate the “Oral Health Values Scale” (OHVS) for use in the Croatian context. **Methods:** The process involved two key phases. First, an expert committee oversaw the translation to ensure consistency across all versions and produce a test-ready instrument. To identify any ambiguities in translation and test the instrument’s reliability, a pilot study with 40 participants was conducted. Once the expert committee confirmed content validity, the finalized OHVS was administered to a sample of 702 Croatian adults to evaluate its psychometric properties. **Results:** Factor analysis revealed a three-factor structure in the Croatian version, differing from the original four-factor model, with items from the “Retaining Natural Teeth” subscale distributed across two factors. Results demonstrated strong internal consistency (Cronbach’s α > 0.78) and test–retest reliability (ICC = 0.976, 95% CI: 0.955–0.987, *p* < 0.01), confirming the instrument’s reliability. **Conclusions:** These results confirm the OHVS-CRO as a valid and reliable instrument for assessing oral health values, offering valuable insights into the perspectives of Croatian populations. This validation study provides a foundation for future research, supports culturally tailored interventions, and highlights the potential for the OHVS to inform oral health research and policy development both locally and globally.

## 1. Introduction

Oral health values can be defined as the extent to which individuals regard their dental health as important, encompassing their commitment to maintaining and enhancing their teeth, gums, and overall orofacial function [1]. These values profoundly influence treatment-seeking behaviors, as individuals who perceive oral health risks as substantial and recognize the benefits of regular dental visits are more likely to maintain consistent oral healthcare [2]. Additionally, oral health values can influence other key behaviors, such as toothbrushing, flossing, smoking and nicotine use, and maintaining a healthy diet [3]. This pattern is particularly evident across different oral healthcare systems: countries with national healthcare integration tend to place a higher perceived value on oral health, whereas those relying on private insurance may reflect different priorities [4]. Preventive care and tooth preservation strategies are widely regarded as public health gold standards, but adherence to these approaches largely depends on how much patients value such care [5].

In Croatia, organized efforts to control and treat dental caries began around 1950, introducing basic preventive measures such as fluoride tablets, local fluoride applications, and regular tooth brushing to promote oral health [6]. From 1968 to 1991, these measures led to a reduction in caries prevalence, with the DMFT (Decayed, Missing, and Filled Teeth) index in 12-year-olds decreasing from 7 to 2.6 [7]. However, this trend reversed in the following years, with the DMFT index rising again to 4.8 by 2009 [8].

Several factors contributed to this shift, including the closure of specialized pediatric dental clinics, insufficient monitoring of dental caries, and a lack of preventive programs [9]. While Croatian adolescents generally report their oral health as very good or excellent, data from the Croatian Central Health Information System show that by 2015, the DMFT index was 4.18 [8,10]. In comparison, global data from 2015 indicate an average DMFT index of 1.86 among 12-year-olds across 209 countries, with 73% of countries reporting averages below this level [11]. The average for European countries stands at 1.81 [12]. This unusual reversal in Croatia’s DMFT trend places the country among those with a high prevalence of caries, underscoring that dental caries remains a significant public health issue [13].

Although oral health values are key to understanding oral health behaviors, they remain underexplored [1]. The closest related construct, which has been extensively researched and is the focus of several validated assessment tools, is oral health-related quality of life (OHRQoL) [14]. OHRQoL is closely linked to oral health values, as both concepts involve perceptions of dental conditions and related aspects [1]. Many health-related quality-of-life measures have been criticized for prioritizing the concerns of clinicians and researchers over what patients value, with some authors suggesting that most OHRQoL measures fail to recognize value systems and lack a comprehensive assessment of the relative importance of orofacial functioning [14,15,16]. Therefore, to truly understand the perception of oral impairments—whether positive or negative—it is essential to examine differences in oral health values [1].

Therefore, in 2021 the Oral Health Values Scale (OHVS) was developed, offering a multidimensional assessment of oral health perceptions. Initially validated with a sample of American participants, the scale comprises items organized into four distinct factors:

Professional Dental Care: this factor addresses the importance of professional dental services, including the associated costs in terms of time, energy, and attention.

Teeth Appearance and Health: this aspect reflects how the appearance and health of teeth contribute to an individual’s pride and highlights the perception of oral health as integral to overall health.

Self-Care Behaviors: the third factor emphasizes the role of dental floss usage as an indicator of consistent self-care practices.

Preservation of Natural Teeth: this factor underscores the value of maintaining natural teeth, linking oral health to functionality and personal well-being [1,17].

The authors of the scale report good reliability for each subscale, with ranges varying from high to acceptable [Professional Dental Care (α = 0.71), Appearance and Health (α = 0.82), Flossing (α = 0.75), and Retaining Natural Teeth (α = 0.72)] [1]. The internal consistency of the questionnaire has been satisfactory in previous studies (α = 0.84, Edwards et al., 2021; α = 0.75, Machado et al., 2022; α = 0.87, Bhadauria et al., 2024) [1,5,18]. As previously mentioned, the original scale comprises a four-factor structure, which has also been observed in the cross-cultural adaptations of the Portuguese and Romanian versions of the scale [5,17]. However, in Bhadauria et al.’s study, a two-factor structure was identified [18].

Understanding oral health values is important not only at the individual level but also for shaping public health strategies across different countries. Namely, cultural differences influence how people prioritize preventive care, adhere to treatments, and seek dental services [19,20,21]. In countries with universal healthcare, preventive measures are highly valued, while in others with privatized systems, curative care may take priority [22]. By creating a Croatian version of the OHVS, this study addresses a gap in international research efforts and facilitates the comparison of oral health values across populations. Therefore, this study aims to develop and validate a Croatian version of the Oral Health Values Scale (OHVS-CRO) as a tool for researching oral health in Croatia. This process includes translating the questionnaire using a double-blind method and evaluating its psychometric properties, such as reliability, response distribution, and factor structure.

## 2. Materials and Methods

The protocol for this study involves a non-experimental cross-sectional survey [23]. Procedures in this section were performed between April and June 2024.

### 2.1. The Investigation of Conceptual and Item Equivalence

For this study, an expert committee was formed to support activities such as translation, psychometric evaluation, and the cross-cultural adaptation process. The committee included a methodologist, two doctors of dental medicine, an English professor, a psychologist, and two native English-speaking translators who participated in the “forward-backward” translation process. To minimize bias, committee members’ identities were disclosed to each other only when direct interaction was required.

Initially, the relationships of the main construct (OHV) in the original and target socio-cultural settings were evaluated. This evaluation included examining the OHVS subscales and items to determine if they were equally relevant and meaningful in the Croatian context. Ensuring conceptual equivalence was a priority, as this would confirm that the construct of oral health values is interpreted consistently across both cultures. To this end, a detailed literature review, discussions with the expert committee, and individualized open interviews were conducted to assess relevance and equivalence. The committee further examined whether any additional subscales or items were needed to fully capture oral health values in the Croatian socio-cultural setting [24,25].

### 2.2. The Investigation of Semantic Equivalence

In the first phase, the instrument was translated into Croatian by two independent translators: one with expertise in dental medicine and knowledge of the concepts being tested and the other without a medical background or familiarity with the concepts. Both translators submitted written reports of their translations.

The next phase was the synthesis of these translations. Based on the synthesized version, two different translators, unaware of the original version, independently translated the questionnaire back into English. The expert committee then harmonized all versions and developed a pre-final version for testing.

The last step of semantic equivalence evaluation involved a pretest. To assess the clarity and understanding of the translated questionnaire, a pilot study was conducted with 40 randomly selected participants. Each participant completed the questionnaire, highlighted unclear words or phrases, and provided comments. Since no changes were needed, a test–retest was conducted a week later on the same sample to evaluate the consistency of responses over time [24,25].

### 2.3. The Investigation of Operational Equivalence

Operational equivalence in this study was achieved through a thorough evaluation by the expert committee, focusing on the pertinence and adequacy of question layout, wording, application setting, and response categorization. This process ensured that the adapted OHVS maintained clarity, cultural relevance, and usability within the Croatian context, supporting reliable data collection and interpretation across cultural lines. The final translated version was evaluated and confirmed by all the members of the expert committee (Supplementary File) [24,25].

### 2.4. Participants and Data Collection

The pilot study was conducted on a random sample of 40 participants from the general population who were invited to complete the paper-based questionnaire in person. Participants were approached in public areas and asked to voluntarily participate in this study [24].

The finalized questionnaire was distributed via an online Google Forms link shared through social networks, email campaigns, and messaging platforms. The link contained an introductory text explaining to the participants the purpose of this study and asking for consent for the data to be used for research purposes, with their confidentiality and anonymity fully protected. The inclusion criteria to participate in this study were as follows: adults aged 18 and over residing in Croatia who were native Croatian speakers. Exclusion criteria included individuals under 18 years of age, non-residents, individuals not fluent in Croatian, and those who did not provide informed consent. To ensure data quality and minimize the risk of multiple entries, participants could only complete the questionnaire once, with access limited to the official survey link. This study adhered to the EU General Data Protection Regulation (GDPR) (Regulation (EU) 2016/679) concerning the protection of personal data [17,26].

### 2.5. Research Materials

The questionnaire consisted of two sections: the first section gathered demographic and general information relevant to the study, including gender, age, place of residence, smoking habits, oral hygiene practices, and employment status. The second section included statements from the translated version of the Oral Health Values Scale (OHVS). The OHVS consists of 12 statements divided into four areas: professional dental care (items 4, 8, 11), appearance and health (items 3, 7, 12), flossing (items 2, 5, 10), and retaining natural teeth (items 1, 6, 9). For the total OHVS score, statements 2, 4, 6, 9, and 11 are reverse-scored. According to the original instrument, respondents rated each statement on a five-point Likert scale (1 = strongly disagree, 2 = disagree, 3 = neutral, 4 = agree, 5 = strongly agree). The total score is calculated by summing all the items, with a theoretical range of 12 to 60 points [1].

### 2.6. Data Analysis

For statistical analysis, IBM SPSS 24 software (version 24.0, SPSS Inc., Chicago, IL, USA) was used. Numerical data were described with percentages, and the mean was presented along with standard deviation. Before analysis, the sample was checked for normal distribution using the Kolmogorov–Smirnov test, which indicated deviation from normality. Since this test can be unreliable for large samples (as it is sensitive to deviations), confidence intervals (CIs) were recommended. The bootstrap method was used to generate confidence intervals, providing theoretical ranges within which the true parameter is expected to reside with 95% confidence [27]. A *t*-test was conducted to compare two categories, while one-way ANOVA was applied for comparisons involving three or more categories. Cronbach’s alpha test was used for reliability testing, and the intraclass correlation coefficient was used to test test–retest reliability [28]. Exploratory factor analysis with oblimin rotation was performed to examine the factor structure. The significance level was set at *p* = 0.01 to minimize the risk of type I errors due to the large sample size and the exploratory nature of the analyses, particularly the factor analysis [27,29].

### 2.7. Ethical Considerations

Written permission to translate and use the OHVS was granted by its author, Cameron L. Randall, PhD, of the University of Washington School of Dentistry, on 21 December 2023. This study was conducted in compliance with the Declaration of Helsinki (1975, revised in 2013) and received approval from the Ethics Committee of the Faculty of Dental Medicine and Health in Osijek (Approval No. 2158/97-97-10-24-12). Participants’ anonymity was guaranteed, making it impossible to identify them based on their responses [30]. A unique code was used in the test–retest phase to ensure anonymity. Written and informed consent was obtained from each participant before proceeding with this study. Participation was voluntary, and participants had the right to withdraw at any time without any negative consequences.

## 3. Results

### 3.1. Pilot Study

A pilot study was conducted prior to the main research. During the pilot study, participants completed the OHVS questionnaire twice: once during the initial assessment and again a week later. This method was used to identify any relevant unclarities and inconsistencies. As no changes were made, this sample of participants was invited to complete the same test, one week later, for retesting purposes. A total of 40 participants took part in the pilot study, of whom 23 were men (58%) and 17 were women (42%), with a median birth year of 1983 for men and 1980 for women.

To assess reliability, we used Cronbach’s alpha test. The overall reliability of the OHVS questionnaire during the first measurement was satisfactory (α = 0.829). The subscale “Retaining Natural Teeth” showed slightly lower reliability (α = 0.527). The overall reliability of the OHVS at the second measurement was also satisfactory (α = 0.883), as well as in all subscales. The difference in reliability in the “Retaining Natural Teeth” subscale can be attributed to the small sample size (Table 1).

Finally, test–retest reliability was assessed. The intraclass correlation coefficient was satisfactory for the overall OHVS score (α = 0.976, 95% CI: 0.955; 0.987, *p* < 0.01) and for all subscales (Table 1).

### 3.2. Study Conducted on the General Population

#### 3.2.1. Descriptive Analysis

This study included 702 participants, the majority of whom were women (68.7%; n = 482), with an average age of 37.93 (±15.23) years. Most participants were employed (66.8%; n = 469) and non-smokers (65.2%; n = 458). A significant portion of the participants were from urban areas (59.25%; n = 416). The majority lived in households with four members. In terms of oral hygiene habits, the largest percentage of participants brushed their teeth twice a day (60.26%). A summary of the sample characteristics is presented in Table 2.

Looking at the overall OHVS results, certain differences among various sample characteristics are evident. Women (M = 46.84 ± 7.36) have higher values for oral health compared to men (M = 43.90 ± 7.99; t = −4.77, *p* < 0.01). Employed participants (M = 46.76 ± 7.54) also show higher oral health values than unemployed individuals (M = 44.2 ± 7.70; t = −4.14, *p* < 0.01). No significant differences in oral health values were found between smokers and non-smokers.

Examining differences based on living location (F(2.699) = 4.07, *p* = 0.02), participants residing in urban areas have higher oral health values compared to those living in rural areas (M = 44.63 ± 7.47; t = 2.91, *p* < 0.001).

Analysis of education levels reveals significant differences in oral health values (F(4.697) = 6.75, *p* < 0.001). A detailed analysis shows a trend: as education level increases, participants assign higher value to oral health. Differences are observed between those with elementary education (M = 40.79 ± 5.88) and all higher education levels: high school (M = 45.19 ± 7.82; t = 2.95, *p* < 0.001), bachelor’s (M = 46.17 ± 7.34; t = −3.74, *p* < 0.001), master’s (M = 47.52 ± 7.46; t = −4.62, *p* < 0.001), and doctoral (M = 48.34 ± 7.93; t = −4.12, *p* < 0.001) (Table 3.).

Items V1 [4.52 (1.10)] and V3 [4.53 (0.89)] received the highest average ratings, while items V2 [3.22 (1.22)] and V10 [2.76 (1.28)] had the lowest scores. The range of results obtained in this study was from 16 to 60 points (Table 4).

#### 3.2.2. Construct Validity

Factor analysis was performed on the questionnaire items to explore its factor structure. The Kaiser–Meyer–Olkin (KMO) measure verified sampling adequacy for factor analysis, yielding a value of 0.834, while Bartlett’s test of sphericity confirmed suitability with χ^2^(66) = 3408.21, *p* < 0.001. Confirmatory factor analysis was then performed to assess the fit of the four-factor structure for the Croatian version of the questionnaire, resulting in the extraction of four factors.

**Figure 1:**
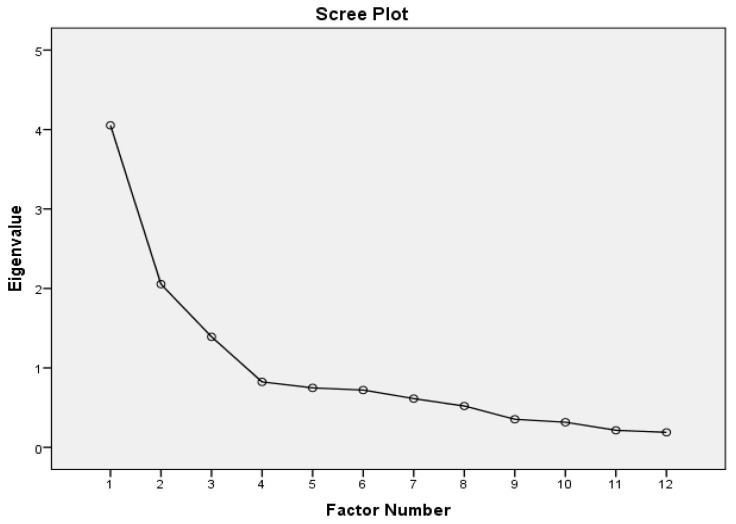
Cattell’s scree plot for determining the distribution of items from the OHVS-CRO questionnaire across subscales.

Communalities analysis indicated that some items fell below the recommended threshold of 0.300, indicating a low percentage of variance explained by the common factors. Items 2, 9, and 11 exhibited communalities less than 0.300 but were retained in the analysis due to their significant contribution to the theoretical model. These items are crucial for understanding the construct being measured, and their removal would undermine the content validity of the instrument. Finally, Cattell’s scree plot indicated the presence of three factors (Figure 1).

The four-factor structure explained 52% of the total variance, which is lower than what would be achieved with a three-factor structure. The issue lies with the last, fourth factor, which has insufficient loading. This final factor contributes only 1.378% to the overall variance explained (Table 5).

The distribution of items across factors does not align with previous studies (Table 6). The items do not group as anticipated based on the original research. A key issue is item V2, which, according to its factor loading, appears to belong to the third factor. However, due to the instruction to “force it to 4”, it was assigned to the fourth factor. Item V1 also has a similar loading to the last factor but actually belongs to factor 1.

In the second step of the factor analysis, exploratory factor analysis with oblique rotation (oblimin) was applied. To assess the suitability for conducting factor analysis, the Kaiser–Meyer–Olkin (KMO) measure yielded a value of 0.83, and Bartlett’s test of sphericity showed χ^2^(702) = 3408.21, *p* < 0.001. These results confirm that the conditions for performing factor analysis are met.

Using the eigenvalue criterion, three factors were extracted, collectively explaining 62.49% of the variance. Factor 1 accounts for 33.77%, Factor 2 for 17.12%, and Factor 3 for 11.59% (Table 7).

The obtained factor structure differs from those in previous studies. Below is a presentation of the factor loadings for each factor (3, 4, 14) (Table 8).

#### 3.2.3. Reliability

To assess the reliability of the questionnaire and its factors, the Cronbach’s alpha coefficient for internal consistency was used. The reliability of the instrument ranges from low to high across the individual factors. Factor 1 demonstrates excellent reliability with a Cronbach’s alpha of 0.92, while Factor 2 has a lower but still acceptable reliability of 0.63. Factor 3 shows a reliability of 0.68, indicating a moderate level of internal consistency. The overall reliability of the Croatian version of the OHVS (OHVS-CRO) is 0.78, which is considered satisfactory. These results suggest that the overall instrument, as well as each factor, maintains an adequate level of reliability.

#### 3.2.4. Relationships Between OHVS Components

Using Spearman’s correlation coefficient, we examined the inter-item correlations for the OHVS-CRO scale and identified a substantial number of significant relationships, with 44 out of 66 correlations reaching statistical significance (Table 9).

We also assessed the correlations between the subscales, finding significant associations among them. Specifically, Factor 1 and Factor 2 showed a moderate positive correlation (r = 0.23, *p* < 0.01), while Factor 1 and Factor 3 exhibited a weak negative correlation (r = −0.18, *p* < 0.01). Additionally, Factor 2 and Factor 3 demonstrated a moderate negative correlation (r = −0.407, *p* < 0.01) (Table 10.). These findings suggest that, while the factors are related, they capture distinct dimensions of the construct.

## 4. Discussion

This study focused on the translation of the Croatian version of the Oral Health Value Scale (OHVS) and the assessment of its validity and reliability in the adult population of the Republic of Croatia. The translation and adaptation of this instrument enables a better evaluation of the importance individuals attribute to oral health. Instruments like OHVS, originally developed in a foreign language, must undergo standard validation protocols before being used in the Croatian-speaking area. This process involves a series of validation protocols, including forward–backward translation and the evaluation of content, criterion, and construct validity to ensure the instrument accurately measures what it intends to [24,25,31].

To date, besides the original version validated in the United States, previous validations and inter-cultural adaptations have been conducted in Romania, Portugal, and India, highlighting its cross-cultural applicability [1,5,17,18]. In the Croatian version, the exploratory factor analysis revealed three key factors, differing from the four-factor model found in the US, Romania, and Portugal, as well as the two-factor structure observed in India [1,5,17,18]. Confirmatory factor analysis further supported the incompatibility of the four-factor structure, with evidence of double loading of some items across three dimensions. The factor analysis led to a recommendation to associate these items to factors with higher loadings, taking into account the content validity of the instrument [32]. Additionally, in the three-factor model, the original dimensions of the questionnaire were merged, with “Appearance and Health” and “Retaining Natural Teeth” combined into a single factor named “Aesthetic Integrity and Teeth Preservation”, while “Professional Dental Care” and “Flossing” remained distinct.

Regarding internal reliability, the Croatian version of the OHVS demonstrated good consistency, particularly in the “Aesthetic Integrity and Teeth Preservation” subscale, which showed excellent reliability. The “Flossing” and “Professional Dental Care” subscales showed acceptable reliability, similar to findings from Romania, where these subscales exhibited varying levels of internal consistency, potentially due to the small number of items in the respective subscales [17].

The comparison of average scores for the items and subscales in the current study with those from the American and Romanian samples revealed similar patterns, with the “Flossing” subscale consistently achieving the lowest scores [1,17]. This suggests that respondents place insufficient emphasis on flossing, despite its critical role in maintaining oral hygiene [33]. In contrast, the “Teeth Health and Aesthetic Integrity” subscale received higher scores, reflecting the widespread perception that oral health is integral not just for aesthetic reasons but for maintaining overall health. This is consistent with findings from a study conducted in Portugal among patients with oral carcinoma, where, despite recognizing its importance to well-being, barriers such as limited accessibility, lack of incentives, and insufficient literacy hinder the adoption of flossing as an integral part of oral hygiene [34,35]. In contrast, a study conducted in India modified the “Flossing” subscale by replacing items related to flossing with those focused on tooth brushing, reflecting cultural differences in oral hygiene practices [18]. These findings underscore the need for enhanced educational initiatives that emphasize the crucial role of flossing in preventing oral diseases and promoting systemic health. Bridging this knowledge gap and incorporating flossing into general oral hygiene education is essential for fostering both short- and long-term health benefits [36,37,38].

The ratio of women to men in this study is similar to research conducted in the United States, Romania, and Portugal, with a slightly higher proportion of women [1,5,17]. This can be attributed to data suggesting that women are generally more health-conscious and proactive in preventive care [39]. Women also tend to be more responsive to questionnaires aimed at enhancing self-awareness [17,40]. The literature also shows that women experience higher levels of dental anxiety, which leads them to visit dental offices more frequently and have a more positive attitude toward dental hygiene [40]. Some studies indicate that in addition to flossing more frequently than men, women also have greater confidence in its effectiveness [33,41]. This pattern was noticeable in this study as well, with results showing that women place higher value on oral health than men. A similar trend was observed in a study on the Romanian population [17]. These findings confirm previous data indicating that women invest more in professional care and appearance and trust the effectiveness of flossing [42]. Although men and women might have the same level of knowledge about oral health, their attitudes and behaviors differ, with women investing more in their appearance and beauty, leading them to take better care of their teeth, follow medical advice, and attend regular check-ups [40]. Research conducted in Romania shows that women take more measures regarding their oral health because they are more concerned about it [17]. This pattern highlights the importance of tailored oral hygiene education to address these gender-specific differences [40]. For women, their smile is a key reason for seeking oral health services [43]. This is further supported by research conducted in Portugal, which highlights a higher value placed on oral health among women [35].

Health values, as assessed by the OHVS, play an important role in shaping individual oral health behaviors and perceptions, which ultimately influence oral health-related quality of life (OHRQoL) [1,44]. In Croatia, significant contributions have been made in the field of OHRQoL research, starting with the work of Petričević et al. [45], who conducted the first cross-cultural adaptation and validation of the Oral Health Impact Profile (OHIP) questionnaire for the Croatian population. This foundational work established a reliable tool for measuring the subjective impact of oral health on daily functioning and has since been widely applied across various population groups. Subsequent studies have explored OHRQoL in different contexts, including university students [46], highlighting the relationship between education level and perceptions of oral health. In studies involving elderly populations, findings have demonstrated that sociodemographic factors, denture use, and subjective health perceptions all contribute to variations in OHRQoL [47]. Recently, the impact of periodontal diseases on OHRQoL was investigated [48]. In addition to studies focused on quality of life, research on oral health knowledge and behaviors demonstrates an inconsistency among participants’ perception and actual oral health status [49]. Aforementioned findings reinforce the need to assess the underlying values and motivations that drive oral health behaviors, as knowledge alone may not necessarily translate into optimal habits. In that context, the OHVS provides a new perspective on oral health by assessing the motivations and values behind these behaviors. This approach bridges the gap between knowledge, practices, and outcomes, making it relevant to conduct future OHVS studies across different Croatian population groups.

One of the primary strengths of this instrument is its relatively brief completion time, which enhances response rates and makes it well suited for clinical applications where time is often a constraint [50]. Despite these advantages, certain limitations must be noted. The probabilistic sampling method employed may reduce the external validity of the findings, as it may not fully represent the diversity of the broader population [51]. Furthermore, the cross-sectional design restricts the ability to conduct longitudinal analyses, which limits the exploration of changes over time and the potential for deeper investigation into the dimensionality of the instrument [52,53]. Additionally, although the study captures a broad age range, the average age of 37.93 years may create a bias toward the behaviors and health attitudes of middle-aged adults [54].

These limitations open opportunities for future research. Future studies could seek to validate the instrument across more targeted populations, such as adolescents or older adults, to broaden its applicability. Furthermore, the inclusion of additional data, such as the frequency of dental visits, could provide a more in-depth understanding of the influences on oral health values. The continued development of more refined instruments for assessing the perceived value of oral health could contribute to comprehensive epidemiological research and interventions aimed at improving related oral health behaviors. By increasing the value placed on oral health, there can be a positive impact on associated behaviors such as self-care and seeking professional dental services [1].

The practical implications of this study’s findings lie in understanding the values that shape oral health behaviors in the Croatian population. These can be integrated into public health initiatives by aligning interventions with the motivations of different groups. For example, emphasizing the value of preserving natural teeth can support campaigns promoting regular dental check-ups and preventive care, while addressing the subscales related to professional dental care and self-care behaviors can guide community education programs aimed at improving oral hygiene practices. Although this study focuses on the Croatian population, its findings have regional and international relevance. The OHVS-CRO adds to research on how oral health values shape behaviors in Central Europe and can support cross-cultural comparisons. By using a consistent framework, future studies can adapt the OHVS in different countries to develop culturally appropriate public health strategies and improve preventive care worldwide.

In conclusion, the successful adaptation of the Oral Health Value Scale (OHVS) to the Croatian language and cultural context marks a significant contribution to advancing oral health research in Croatia. The OHVS-CRO demonstrates robust psychometric properties, with three relevant factors that capture the cultural perceptions and values related to oral health in the Croatian population. While the scale has proven reliable and valid, further investigation is needed to assess its applicability across diverse demographic groups. Additionally, incorporating additional variables would enhance the scale’s comprehensiveness, enabling more nuanced insights into oral health behaviors and facilitating the development of targeted interventions. This adaptation not only expands the reach of oral health research within Croatia but also lays a crucial foundation for further cross-cultural validations, ultimately contributing to the formulation of more precise and effective oral health policies and practices.

## Figures and Tables

**Table 1:** Reliability of OHVS-CRO subscales in both measurements.

	1st Measurement	2nd Measurement	ICC	Interclass Correlation Coefficient ICC (95%)	*p* *
	Reliability	Reliability			
OHVS—total	0.829	0.883	0.976	0.955, 0.987	<0.00
Professional dental care	0.674	0.663	0.933	0.874, 0.965	<0.01
Appearance and health	0.733	0.908	0.928	0.864, 0.962	<0.01
Flossing	0.703	0.770	0.950	0.906, 0.974	<0.01
Retaining natural teeth	0.527	0.653	0.949	0.903, 0.973	<0.01

ICC—interclass correlation coefficient; * *p*-value = 0.005.

**Table 2:** Distribution of demographic variables in the sample.

Participant Characteristics		N	%
Gender	Male	220	31.34
	Female	482	68.66
Residence	Village	197	28.06
	Suburb	89	12.68
	City	416	59.26
Education	Elementary	29	4.13
	High school	315	44.87
	Bachelor’s degree	162	23.08
	Master’s degree	167	23.79
	Doctoral degree	29	4.13
Employed	Yes	469	66.81
	No	233	33.19
Smoking	Yes	244	34.76
	No	458	65.24
Oral hygiene habits	0	7	0.01
	1	76	10.83
	2	423	60.26
	3	176	25.07
	4	14	0.02
	5	4	0.01
	6	1	0.00
	9	1	0.00
	Total sample	702	100

**Table 3:** Comparison of OHVS results by category.

		N	M	SD	T	*p*
Gender	Male	220	43.90	7.99	−4.77	0.00 **
	Female	482	46.84	7.36		
Employed	Yes	469	46.76	7.54	−4.14	0.00 **
	No	233	44.24	7.70		
Smoking	Yes	244	45.82	7.95	0.27	0.79
	No	458	45.98	7.55		
		N	M	SD	F	*p*
Residence	Village	197	44.63	7.47	4.07	0.02 *
	Suburb	89	46.01	8.61		
	City	416	46.51	7.51		
Education	Elementary	29	40.79	5.88	6.75	0.00 **
	High school	315	45.19	7.82		
	Bachelor’s degree	162	46.17	7.34		
	Master’s degree	167	47.52	7.46		
	Doctoral degree	29	48.34	7.93		

N—number of participants; M—mean; SD—standard deviation; T—*t*-test; *p*—one-way analysis of variance; ** *p* < 0.01 * *p* < 0.05.

**Table 4:** Differences in the total score of OHVS-CRO and its individual items.

	M (SD)	Median (IQR)	Min–Max
Item 1	4.52 (1.10)	5.00 (0.00)	1–5
Item 2	3.22 (1.22)	3.00 (2.00)	1–5
Item 3	4.35 (1.10)	5.00 (1.00)	1–5
Item 4	4.14 (1.16)	5.00 (1.00)	1–5
Item 5	3.32 (1.16)	3.00 (1.00)	1–5
Item 6	4.53 (0.89)	5.00 (1.00)	1–5
Item 7	4.19 (1.17)	5.00 (1.00)	1–5
Item 8	3.64 (1.26)	4.00 (2.00)	1–5
Item 9	3.54 (1.23)	4.00 (2.00)	1–5
Item 10	2.76 (1.28)	3.00 (2.00)	1–5
Item 11	3.36 (1.38)	4.00 (3.00)	1–5
Item 12	4.36 (1.12)	5.00 (1.00)	1–5
OHVS-CRO total score	45.92 (7.68)	46.00 (12.00)	16–60

M—mean; SD—standard deviation; IQR—interquartile range; Min—minimum achieved value; Max—maximum achieved value.

**Table 5:** Variance distribution of the OHVS scale across the four extracted factors.

Factor	Extraction Sums of Squared Loadings		
	Total	% of Variance	Cumulative %
Factor 1	3.76	31.29	31.29
Factor 2	1.52	12.66	43.95
Factor 3	0.87	7.29	51.24
Factor 4	0.17	1.38	52.61

**Table 6:** Distribution of items across factors in the four-factor structure.

Factor 1	Factor 2	Factor 3	Factor 4
Item 3	Item 6	Item 10	Item 2
Item 7	Item 4	Item 5	
Item 12	Item 8		
Item 1	Item 11		
	Item 9		

**Table 7:** Variance distribution of the OHVS scale across the extracted factors.

Factor	Extraction Sums of Squared Loadings		
	Total	% of Variance	Cumulative %
Factor 1	4.053	33.76	33.76
Factor 2	2.054	17.12	50.89
Factor 3	1.391	11.59	62.49

**Table 8:** Assignment of items to factors with information on factor loadings.

Item	Factor 1	Factor 2	Factor 3
3	0.89		
1	0.89		
12	0.89		
7	0.83		
4		0.70	
9		0.62	
6	0.39	0.61	
11		0.57	
8		0.60	0.34
10			0.85
5			0.77
2			0.61

**Table 9:** Correlation between OHVS-CRO items.

	V1	V2	V3	V4	V5	V6	V7	V8	V9	V10	V11	V12
V1	1	−0.06	0.74 **	0.18 **	0.33 **	0.37 **	0.68 **	0.06	0.08 *	0.16 **	−0.07	0.77 **
V2		1	0.00	0.12 **	0.31 *	0.12 **	0.07	0.23 **	0.13 **	0.29 **	0.25 **	−0.01
V3		0.00	1	0.14 **	0.38 **	0.38 **	0.79 **	0.09 *	0.09 *	0.24 **	0.01	0.77 **
V4				1	0.15 **	0.38 **	0.16 **	0.27 **	0.22 **	0.06	0.23 **	0.16 **
V5					1	0.20 **	0.41 **	0.28 **	0.08 *	0.63 **	0.15 **	0.37 **
V6						1	0.35 **	0.29 **	0.22 **	0.11 **	0.21 **	0.40 **
V7							1	0.09 *	0.14 **	0.28 **	0.00	0.73 **
V8								1	0.29 **	0.31 **	0.32 **	0.14 **
V9									1	.06	0.19 **	0.12 **
V10										1	0.14 **	0.23 **
V11											1	−0.01
V12												1

** *p* < 0.01; * *p* < 0.05.

**Table 10:** Correlations between OHVS-CRO subscales.

	Factor 1	Factor 2	Factor 3
Factor1	1	0.23 **	−0.18 **
Factor2		1	−0.407 **
Factor 3			1

** *p* < 0.01.

## Data Availability

The original contributions presented in this study are included in the article and Supplementary Files. Further inquiries can be directed to the corresponding authors.

## Supplementary Materials

The following supporting information can be downloaded at: https://www.mdpi.com/article/10.3390/dj13020056/s1. Table S1. The translation of OHVS in Croatian language.
