# Supplementary material for: Psychometric Analysis and Cross-Cultural Adaptation of the Croatian Version of the Oral Health Values Scale (OHVS)
Source: Dent J (Basel). 2025 Jan 27;13(2):56. doi: 10.3390/dj13020056 (PMC11854085; doi:10.3390/dj13020056)
Supplement: Supplementary file 1 [file dentistry-13-00056-s001.zip › dentistry-3410409-supplementary.pdf]

Table S1. The translation of OHVS in Croatian language

Original Oral Health Values Scale	Final translated version
1. It is important to me to keep my natural teeth.	1. Važno mi je zadržati svoje prirodne zube.
2. It is okay for me to miss a day or two of flossing when I am busy.*	2. Kada nemam vremena u redu mi je propustiti dan ili dva dana čišćenja zubnim koncem.*
3. My smile is an important part of my appearance.	3. Moj osmijeh je važan dio mog izgleda.
4. Going to a dentist is not worth the cost to me.*	4. Odlazak stomatologu za mene nije vrijedan novčanog troška.*
5. Flossing my teeth every day is a high priority for me.	5. Svakodnevno čišćenje zuba zubnim koncem za mene je od velike važnosti.
6. I would rather get dentures than spend money to treat cavities or gum disease*	6. Radije bih nosio/la zubnu protezu nego trošio/la novac na liječenje karijesa ili bolesti desni.*
7. I think it is important that my teeth and gums are a source of pride.	7. Važno mi je da se mogu ponositi svojim zubima i desnima.
8. If I have a toothache, I prefer to wait and see if it will go away on its own before seeing a dentist.*	8. Ako me boli zub, prije nego što odem stomatologu, radije ću pričekati i vidjeti hoće li bol nestati sama od sebe.*
9. I would not mind if I had to have a false tooth or dentures.*	9. Ne bi mi smetalo kada bih morao/la imati umjetni zub ili zubnu protezu.*
10. I make sure I have dental floss available with me so I have it when I need it.	10. Vodim računa da kod sebe uvijek imam konac za zube kako bih ga upotrijebio kada mi je potreban.
11. Going to the dentist is only important if my teeth or gums are bothering me.*	11. Važno mi je otići stomatologu samo ako imam problema sa zubima ili desnima.*
12. The condition of my teeth and gums is an important part of my overall health.	12. Stanje mojih zubi i desni važan je dio mog cjelokupnog zdravlja.
Note: *Denotes items that are reverse scored. Profesional Dental Care factor/subscale: items 4,8,11; Apperance and Health factor/subscale: items 3,7,12; Flossing factor/subscale: items 2,5,10; Retaining Natural Teeth factor/subscale: Items 1,6,9.	
